# Microarray comparison of prostate tumor gene expression in African-American and Caucasian American males: a pilot project study

**DOI:** 10.1186/1750-9378-4-S1-S3

**Published:** 2009-02-10

**Authors:** R Renee Reams, Deepak Agrawal, Melissa B Davis, Sean Yoder, Folakemi T Odedina, Nagi Kumar, Joseph M Higginbotham, Titilola Akinremi, Sandra Suther, Karam FA Soliman

**Affiliations:** 1 College of Pharmacy & Pharmaceutical Sciences Florida A&M University, Tallahassee, Florida, USA; 2 H. Lee Moffitt Cancer Center Tampa, Florida, USA; 3 Institute of Genomics and Systems Biology, The University of Chicago, Chicago, Illinois, USA; 4 Department of Pathology, Federal Medical Center (FMC), Abeokuta, Nigeria; 5 Institute of Public Health, Florida A&M University, Tallahassee, Florida, USA

## Abstract

African American Men are 65% more likely to develop prostate cancer and are twice as likely to die of prostate cancer, than are Caucasian American Males. The explanation for this glaring health disparity is still unknown; although a number of different plausible factors have been offered including genetic susceptibility and gene-environment interactions. We favor the hypothesis that altered gene expression plays a major role in the disparity observed in prostate cancer incidence and mortality between African American and Caucasian American Males. To discover genes or gene expression pattern(s) unique to African American or to Caucasian American Males that explain the observed prostate cancer health disparity in African American males, we conducted a micro array pilot project study that used prostate tumors with a Gleason score of 6. We compared gene expression profiling in tumors from African-American Males to prostate tumors in Caucasian American Males. A comparison of case-matched ratios revealed at least 67 statistically significant genes that met filtering criteria of at least +/- 4.0 fold change and p < 0.0001. Gene ontology terms prevalent in African American prostate tumor/normal ratios relative to Caucasian American prostate tumor/normal ratios included interleukins, progesterone signaling, Chromatin-mediated maintenance and myeloid dendritic cell proliferation. Functional in vitro assays are underway to determine roles that selected genes in these onotologies play in contributing to prostate cancer development and health disparity.

## Introduction

African American males (AAM) have a 1.6 increased risk of developing prostate cancer (PCa), and a 2.5 fold increased mortality rate relative to Caucasian American males (CAM) [1] The explanation for this greater PCa burden in African Americans, still unclear, is probably multi-factorial and may include diet, access to care, racial differences in PCa treatment, socioeconomic status, attitudes about care and or differences in gene expression or gene-environment [2-4]. The aim of this pilot project study is to uncover gene expression differences that explain PCa health disparity in AAM.

## Methods

### Acquisition of samples

This pilot project study was conducted using snap frozen PCa tumors and non-tumor matched controls. Authorization for tumor tissue used in this study was obtained through IRB approved protocols from Florida A&M University and from the H. Lee Moffitt Cancer Center. Selection of prostate tissue from the Moffitt Cancer Center Tissue Bank was made based on race/ethnicity, availability of tumors with same Gleason grade/score. Due to the limited tissue availability, this pilot project used a total of 16 prostate tissue samples. We used four snap frozen PCa tumors and four matched controls from CAM men and four snap frozen PCa tumors and four matched controls from AAM. All PCa tumors in this study had a Gleason score of 3+3 = 6. The 3+3 designation refers to the histological pattern or grade of the prostate cells. For example, if 95% or more of the tumor is composed of one pattern, the corresponding number is counted twice, thus an entire moderately differentiated tumor would be scored as 3+3 = 6. A Gleason score of 2–4 is low grade, 5–7 is intermediate grade and 8–10 is high grade.

### RNA isolation, probe preparation, hybridization and scans

Total RNA from excised tissue specimens was isolated using the TRIzol Reagent (Invitrogen, Carlsbad, CA) and the manufacturer's protocol. We prepared, labeled RNA Targets for hybridization using protocols initially described by Van Gelder et al. [5] Probe arrays were scanned once at 1.5-μm resolution using the Affymetrix Gene Chip Scanner 3000 at the Moffitt Cancer Institute.

### Micro array data

Gene expression profiles were measured for each of the micro dissected prostate tissue samples using the Affymetrix U133A human arrays. Each of the 8 prostate tumors and 8 matched control samples underwent single hybridization and was arrayed individually (i.e. samples were not pooled). Data from the micro array CEL files was uploaded to R-bioconductor for analysis. Gene changes were then selected using the Significance Analysis of Micro arrays (SAM) technique of Tusher et al. [6]. We paired normal tissue samples with the tumor samples from the same individuals for each race group. The results of these case paired t-tests are described below.

## Results

### Results of the CAM case paired t-test

**Figure 1:**
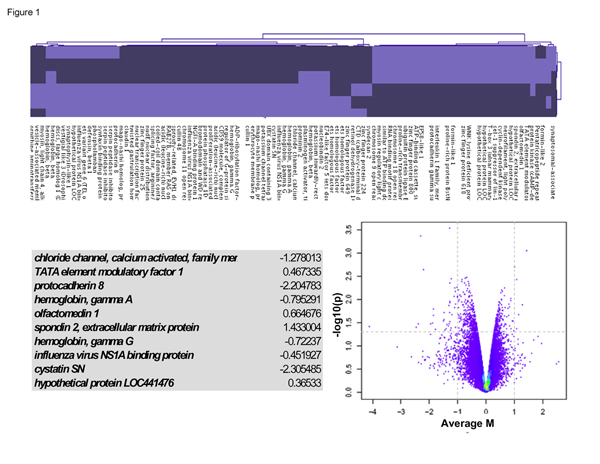
**Caucasian tumor vs. normal: Case-paired t-test analysis**. A comparison of micro array data of tumor versus normal for prostate sample from CAM was analyzed using R-bioconductor.

Approximately 100 genes clustered to give a distinct signal indicating a subset of genes that were up regulated in CAM prostate tumors (see lighter pattern in cluster gram, Figure 1) but down-regulated (see darker pattern in cluster gram, Figure 1) in normal tissue of CAM. Genes listed in the cluster gram in Figure 1 included protocadherin 8 (down regulated 2.2 fold) and Cystatin SN (down regulated 2.3 fold). However, the filtering criteria of 4.0 fold change and p < 0.0001 were not met for any of the 100 genes that resulted from this paired t-test; hence these genes will not be discussed. In the Volcano plot on lower right side of Figure 1 the x-axis denotes fold change (a minus 2 value represents a negative 2.0 fold change) and y axis denotes minus the log of p-value; hence a y-axis value of 1.0, 2.0 and 3.0 represents a p-value of 0.01, 0.001 and 0.0001 respectively).

### Results of the AAM case paired t-test

**Figure 2:**
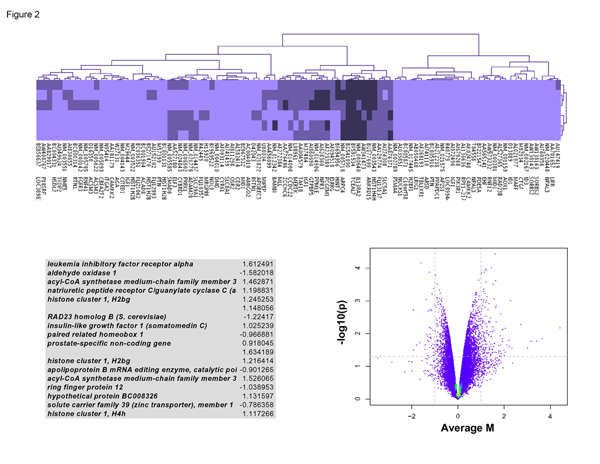
**African American male tumor vs. normal: case-paired t-test analysis**. A comparison of micro array data from tumor versus normal for the prostate sample from African American (AA) Males was analyzed using R-bioconductor. This figure shows (1) a cluster gram of differentially expressed genes in the three prostate tumor sample vs. the three matching non-tumor prostate tissue in African American males; (2) an MA plot is also shown in the lower left of this figure and (3) a Volcano Plot depicting additional information about these differentially expressed genes is shown in the lower right of this figure. The cluster gram shows a high degree of variation from among AA tumor samples and among AA normal samples. The Volcano and MA plots both showed an absence of high p-values and an absence of high fold change.

The raw data was reduced to a list of 100 genes, represented in the cluster gram in Figure 2. Pairing normal tissue samples with the tumor samples for African American Males, we observed a high degree of genetic variance among tumor samples and non-tumor samples. Hence, it was impossible to find differentially expressed genes that met the filtering criteria of 4.0 fold change and p < 0.0001.

### Overlap of case paired t-test for AAM & CAM gene lists

**Figure 3:**
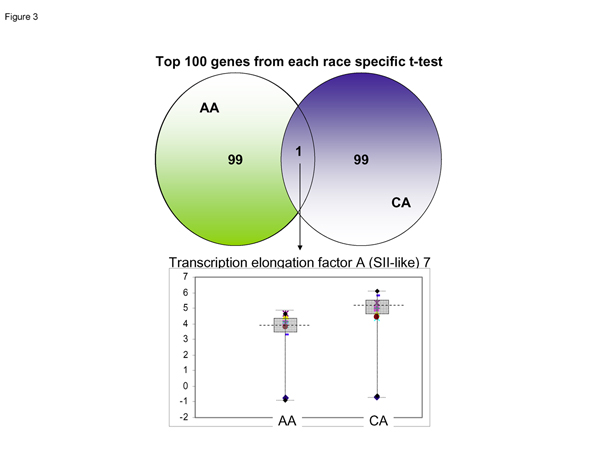
**Overlaps of case-paired t-test for African American male (tumor vs. normal) and Caucasian American Males (tumor vs. normal) gene lists**. A comparison of the top 100 genes from the CA tumor vs. normal gene list to the top 100 genes from the AA tumor vs. normal top 100 genes revealed that there was only one gene common to both these gene lists. That gene is TCEAL, a transcription elongation factor A-like 7 gene.

Figure 3 shows a comparison of the top 100 genes from the AAM profile to the top 100 genes from CAM profiles showed only one gene common between these differentially expressed gene lists; that gene was a Transcription Elongation Factor A (SII) like-7 gene proteins (TCEAL 7).

### Case matched ratios-race group test

**Figure 4:**
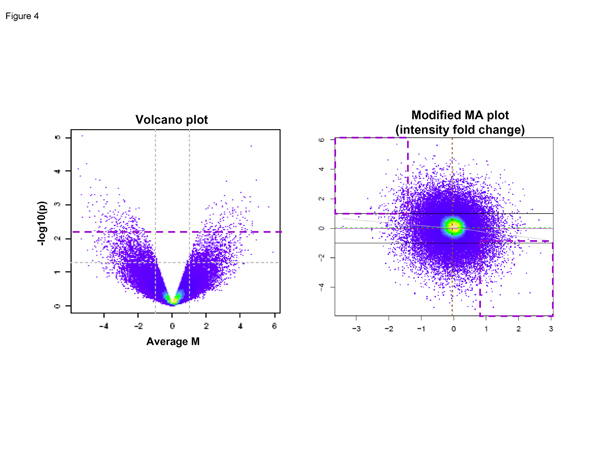
**Case-matched ratios-race group tests**. Figure 4 depicts a Volcano plot on the left and a Modified MA plot on the right. The volcano plot shows the presence of genes (represented by dots) that have high gene expression profile changes and that are also highly statistically significant as evidenced by the p-values and the large fold change values (genes of interest fall above the dashed bold line). On the volcano plot, the x-axis denotes fold change of the Caucasian samples (i.e., a minus 2 represents a negative 2.0 fold change of tumor vs. normal) and the y axis denotes fold change of the African American samples. The modified MA plot shows fold change on the axis and intensity on the x-axis. The dots in the dashed squares in the upper left hand corner of the MA plot contains genes whose fold change values met our filtering criteria of positive in CA and negative in AA. The lower right dashed square contains genes that met our filtering criteria of positive in AA and negative in CA.

Figure 4 shows the result of comparing AAM case matched ratios to CAM case matched ratios. We successfully controlled for the AAM high degree of genetic variation and were able to identify 67 differentially expressed genes in AAM compared to CAM.

### Case-matched ratios-test for race specific expression trends

**Figure 5:**
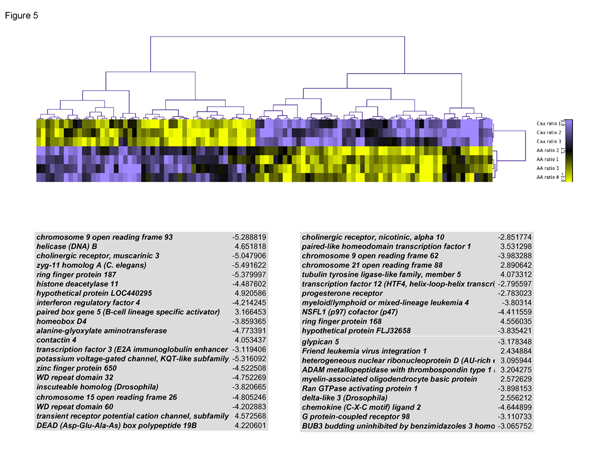
**Case-matched ratios-race group tests for specific expression trends**. A cluster gram is shown in Figure 5. Those genes found to be differentially expressed were clustered using hierarchical cluster analysis to visualize the pattern of gene expression for each sample. Samples which represents the gene expression pattern that results when CA tumor/CA normal ratio is compared to AA tumor/AA Normal. The heat map indicates that blue represents highly expressed genes, while yellow represents under expressed genes; the black color on the heat map represented equal expression relative to control.

Those genes found to be differentially expressed in AAM were clustered using hierarchical cluster analysis to visualize the pattern of gene expression for AAM tumor/normal ratio to the CAM tumor/normal ratio. The key for sample description is shown next to the heat map to the right of the cluster gram, The first three horizontal rows of the cluster gram are designated as CA ratio 1–3 and represent three of the four CAM PCa tumor vs control data sets used in this experiment. The AA ratio 1–4 represents the four AAM PCa tumor vs control data sets used. These clustered genes represented the top 100 genes from our ratios test for race specific gene expression trends (highest p-value observed was 9.28 E-06; lowest p-value observed 0.002). The cluster gram in Figure 5 showed that genes up regulated in AAM are simultaneously down regulated in CAM.

### Gene ontology enrichment

Table 1 shows the significant gene ontology terms affected in AAM tumor/normal case matched ratios relative to CAM tumor/normal case matched ratios. Terms most prevalent among differentially expressed genes in AAM are interleukins, chromatin-mediated maintenance, progesterone signaling and myeloid dendritic cell differentiation.

**Table 1:** Gene Ontology Enrichment

**GOBPID**	**Pvalue**	**OddsRat**	**ExpCount**	**Count**	**Size**	**Term**
GO:0042091	0.00468	lnf	0.00468	1	1	interleukin-10 biosynthetic process
GO:0016340	0.00468	Inf	0.00468	1	1	calcium-dependent cell-matrix adhesion
GO:0045082	0.00468	lnf	0.00468	1	1	positive regulation of interleukin-10 biosynthetic process
GO:0045074	0.00468	Inf	0.00468	1	1	regulation of interleukin-10 biosynthetic process
GO:0007275	0.00536	2.23501	9.72558	18	2076	multicellular orgranismal development
GO:0048856	0.00558	2.2656	8.98071	17	1917	anatomical structure development
GO:0006478	0.00935	216.103	0.00937	1	2	peptidyl-tyrosine sulfation
GO:0035054	0.00935	216.103	0.00937	1	2	embryonic heart tube anteriorlposterior pattern formation
GO:0045366	0.00935	216.103	0.00937	1	2	regulation of interleukin-13 biosynthetic process
GO:0045368	0.00935	216.103	0.00937	1	2	positive regulation of interleukin-13 biosynthetic process
GO:0032613	0.00935	216.103	0.00937	1	2	interleukin-10 production
GO:0043283	0.01001	1.9026	20.8238	30	4445	bipolymer metabolic process
GO:0048731	0.01327	2.16651	7.43942	14	1588	system development
GO:0007399	0.01366	2.76046	3.19501	8	682	nervous system development
GO:0048617	0.01399	108.043	0.01405	1	3	embryonic foregut morphogenesis
GO:0042097	0.01399	108.043	0.01405	1	3	interleukin-4 biosynthetic process
GO:0046487	0.01399	108.043	0.01405	1	3	glyoxylate metabolic process
GO:0042231	0.01399	108.043	0.01405	1	3	interleukin-13 biosynthetic process
GO:0006538	0.01399	108.043	0.01405	1	3	glutamate catabolic process
GO:0045402	0.01399	108.043	0.01405	1	3	regulation of interleukin-4 biosynthetic process
GO:0045404	0.01399	108.043	0.01405	1	3	positive regulation of interleukin-4 biosynthetic process
GO:0048096	0.01399	108.043	0.01405	1	3	chromatin-mediated maintenance of transcription
GO:0032616	0.01399	108.043	0.01405	1	3	interleukin-13 production
GO:0050847	0.01399	108.043	0.01405	1	3	progesterone receptor signaling pathway
GO:0048339	0.01399	108.043	0.01405	1	3	paraxial mesoderm development
GO:0043011	0.01399	108.043	0.01405	1	3	myeloid dendritic cell differentiation

## Discussion

The purpose of this pilot project study was to search for genes unique to African American or Caucasian American Males that explain the observed PCa health disparity between the two races. The novel findings of this study resulted in the identification of at least four candidate genes that warrant further investigation as to their role in PCa health disparity. First we observed that *TCEAL 7 *expression in PCa tumor and in non-tumor tissue was higher in CAM compared to AAM. The TCEAL7 gene modulates transcription in a promoter context-dependent manner. Moreover, a recent study [7] has implicated TCEAL 7 as a cell death regulatory protein frequently inactivated by methylation in ovarian cancer. These authors showed that when re-expressed in cancer cell lines, TCEAL 7 induces cell death and reduces colony formation; thus suggesting that TCEAL 7 functions as a tumor suppressor protein. Because TCEAL 7 expression is slightly higher in CAM than in AAM it is interesting to speculate that increased expression of TCEAL 7 in CAM may correlate with increased suppression of prostate tumors in CAM but decreased prostate tumor suppression in AAM.

The second set of novel findings in this study was the identification of gene ontology (GO) terms and pathways that are prevalent in the 67 down-regulated genes (see Table 1). The GO terms most prevalent in AAM were interleukins, progesterone signaling and chromatin-mediated maintenance. Genes associated with these GO ontologies, which were decreased in expression in AAM compared to CAM, were Histone Deacetylase 11 or HDAC 11 (down regulated by 4.4876 fold); Interferon regulatory factor 4 (down regulated by 4.2142 fold), and the progesterone receptor (down regulated by 2.7830 fold). HDAC is an emerging oncology target. HDAC regulates gene expression and overexpressed or sustained HDAC activity, occurring in some cancer cells, results in deacetylation of the histone tails or histone hypoacetylation. Histone hypoacetylation results in more closed or tightly packaged chromatin structure in which certain genes may become inaccessible to transcription factors and hence will not get transcribed. Such events are being studied aggressively as a plausible mechanism for limiting or silencing tumor suppressor genes. We have yet to discover the significance of HDAC 11 down regulation in AAM samples compared to CAM samples in this pilot project study. The finding that the progesterone receptor is down regulated in AAM relative to CAM samples is very intriguing in light of the fact that decreased progesterone receptor expression contributes to poor prognosis for breast cancer in African American women. That this pilot project data underscores the prevalence of interleukins is noteworthy because a report [8] on tumor immunological differences between African Americans and European American Males was published as this data was being summarized. Taken together, this study revealed 67 genes that are differentially expressed in AAM prostate tumors, compared to CAM. Functional assay studies are underway to investigate the role at least four of these genes and the role that they play in prostate cancer development and health disparity.

## Competing interests

The authors declare that they have no competing interests.
